# Knowledge, attitudes, and practices of ICU physicians on antimicrobial use and resistance: a scoping review

**DOI:** 10.1017/ash.2025.10249

**Published:** 2025-12-18

**Authors:** Filipe Teixeira Piastrelli, Giovanna Marssola Nascimento, Haliton Alves de Oliveira Junior, Pablo Kokay Valente, Ícaro Boszczowski

**Affiliations:** 1 Infection Control Department, Hospital Alemão Oswaldo Cruz, São Paulo, Brazil; 2 Rede Américas, Infection Control Department, Hospital Samaritano, São Paulo, Brazil; 3 Health Technology Assessment Center, Hospital Beneficência Portuguesa, São Paulo, Brazil; 4 Department of Allied Health Sciences, University of Connecticut, Storrs, CT, USA; 5 Infection Control Department and Hospital Nove de Julho, Rede Américas, Infection Control Department, Hospital das Clínicas da Faculdade de Medicina da Universidade de São Paulo, São Paulo, Brazil

## Abstract

**Objective:**

To identify behavioral factors explored in the literature and the theoretical frameworks used to understand antimicrobial prescribing behaviors of ICU physicians.

**Design:**

Scoping review following the JBI methodology.

**Setting:**

Studies conducted in intensive care units (ICUs) across various healthcare systems.

**Participants:**

Physicians working in ICUs; studies involving other healthcare professionals or aggregating data from multiple specialties were excluded.

**Interventions:**

Not applicable.

**Results:**

From 995 records identified through PubMed, Embase, Scopus, CINAHL, and Web of Science, 18 studies met inclusion criteria. Fourteen were cross-sectional surveys and four used qualitative semi-structured interviews. Knowledge about antimicrobial use and its role in resistance was generally adequate. Attitudes reflected that beliefs, clinical uncertainty, and contextual factors influenced prescribing behaviors. Reported practices highlighted the role of adherence to institutional protocols and guidelines. Despite the behavioral focus, most studies lacked explicit use of theoretical frameworks to guide data collection or interpretation.

**Conclusions:**

Antimicrobial prescribing in ICUs is influenced by behavioral determinants that are not consistently evaluated using theoretical models. Future research on Knowledge, Attitudes, and Practices (KAP) should integrate behavioral science frameworks to enhance understanding and enable better design of stewardship interventions.

## Introduction

Antimicrobial resistance (AMR) represents a global public health threat, negatively impacting patient clinical outcomes by increasing morbidity, mortality, and hospital costs.^1,2^The fight against AMR was further challenged by the emergence of COVID-19, which saw an increased incidence of resistance during the pandemic period.^3^ Before this, a study estimated that infections caused by drug-resistant microorganisms accounted for approximately 4.95 million deaths in 2019, with one-quarter of this number directly attributable to drug resistance.^4^

The ICUs represent a unique hospital environment, characterized by the highest rates of microbial resistance and density of antimicrobial use. Inadequate initial therapy selection for critically ill patients is associated with worse clinical outcomes, often resulting in higher use of broad-spectrum drugs and therapies, even when risk factors for resistance are not present.^5^ Therefore, Antimicrobial Stewardship Programs (ASP) frequently prioritize ICUs for target interventions.^6^

Antimicrobial prescribing is a complex process influenced by multiple factors, including cultural, environmental, and behavioral determinants.^7^ Studies assessing knowledge, attitudes, and practices (KAP) related to antimicrobial use and bacterial resistance reveal knowledge gaps among physicians and the impact of sociocultural context – such as hierarchy, reputational risk, tolerance of uncertainty, and others – on prescribing behavior.^8–11^

Moreover, behavioral theories and frameworks provide the basis for analysis of the structure and psychological processes that likely regulate behavior. However, these theories and frameworks are not used systematically, which hinders both the comparisons of studies and the development of effective intervention strategies.^12^ There is a need for more comprehensive studies on this topic, particularly in the ICU setting. We focus on intensivists, as they are the primary decision-makers in selecting, adjusting, and discontinuing antimicrobial therapy within intensive care settings. Hence, this scoping review aims to determine which intensivists’ KAP has been evaluated and what frameworks and theories have been applied to map key factors influencing antimicrobial prescribing behavior in ICUs.

## Methods

### Review question

Primary review question: What are the knowledge, attitudes and practices on antimicrobial prescriptions and AMR by physicians in intensive care units?

#### Secondary review question

What behavioral theoretical frameworks are used in quantitative and qualitative research on this topic?

### Inclusion criteria

### Participants

This review included studies examining intensivists’ KAP regarding antibiotic prescribing and AMR. All physicians directly working in the ICU were considered intensivists to inclusion, regardless of their primary medical specialty. Studies that included data from other medical specialties or intensive care professional categories were eligible if the data were presented separately, and only information specific to intensivists was included in the analysis.

### Concept

The concept of interest in this scoping review is the general KAP of intensive care physicians regarding antimicrobial prescribing and AMR. The review specifically aimed to map broad, cross-cutting KAP constructs, such as overall awareness of AMR and general understanding of antimicrobial use, prescribing behaviors, and attitudes.

### Context

Intensive care units (ICU) represent the hospital unit with the highest proportion of antimicrobial use,^13^ although they are underrepresented in studies that evaluate behavioral factors that determine antimicrobial prescription patterns. Furthermore, methodological heterogeneity limited comparability across studies. Despite differences in ICU subspecialties, they share similar prescribing contexts; therefore, this review included adult, pediatric, neonatal, and other ICU types.

### Types of sources

This scoping review considered primary research studies with quantitative, qualitative, and mixed methods study designs. Systematic reviews, narrative reviews and clinical reviews were not included.

We adopted the Joanna Briggs Institute (JBI) methodology for scoping reviews^14^ and the Preferred Reporting Items for Systematic Reviews and Meta-Analyses extension for Scoping Reviews^15^ as follows: develop review questions and review objective; determine the eligibility criteria; develop the literature search strategy; extract, analyze and discuss the findings; draw conclusions and discuss the implications for practice and further research.^14^

### Search strategy

The search strategy aimed to locate both published and unpublished studies. A three-step search strategy was utilized in this review. First, an initial limited search of PubMed. The text words contained in the titles and abstracts of relevant articles, and the index terms used to describe the articles were used to develop a full search strategy. The search strategy, incorporating all identified keywords and index terms, was tailored to each included database and information source, covering searches in English, Spanish, French, and Portuguese. The reference list of all included sources of evidence was screened for additional studies.

The databases searched include PubMed, Embase, Scopus, CINAHL (Cumulative Index to Nursing and Allied Health Literature), and Web of Science databases. Full search strategies for each database are provided in Supplementary File.

### Study/Source of evidence selection

Following the search, all identified citations were collated and uploaded into Mendeley Reference Manager® (version 2.118.0, Elsevier, 2024), and duplicates were removed. Then, titles and abstracts were screened by one reviewer (FTP) for assessment against the inclusion criteria for the review. Potentially relevant sources were retrieved in full text and their citation details were imported into Rayyan® (version 1.5.0, Rayyan Systems, Inc., 2024). The full text of selected citations was assessed in detail against the inclusion criteria by two independent reviewers (GMN and FTP). Reasons for the exclusion of sources of evidence in full text that do not meet the inclusion criteria were recorded and reported. Any disagreements that remained between the reviewers at each stage of the selection process were resolved with a third reviewer (IB).

### Data extraction

Data was manually extracted from each selected study. The data extracted included authors, year, country, type of publication, study design, sample size, validation, study aims, theoretical frameworks, ICU type, measurement methods, quantitative results, qualitative results, conclusion and notes (see Appendix 1 for data extraction tool).

### Results

**Figure 1.**
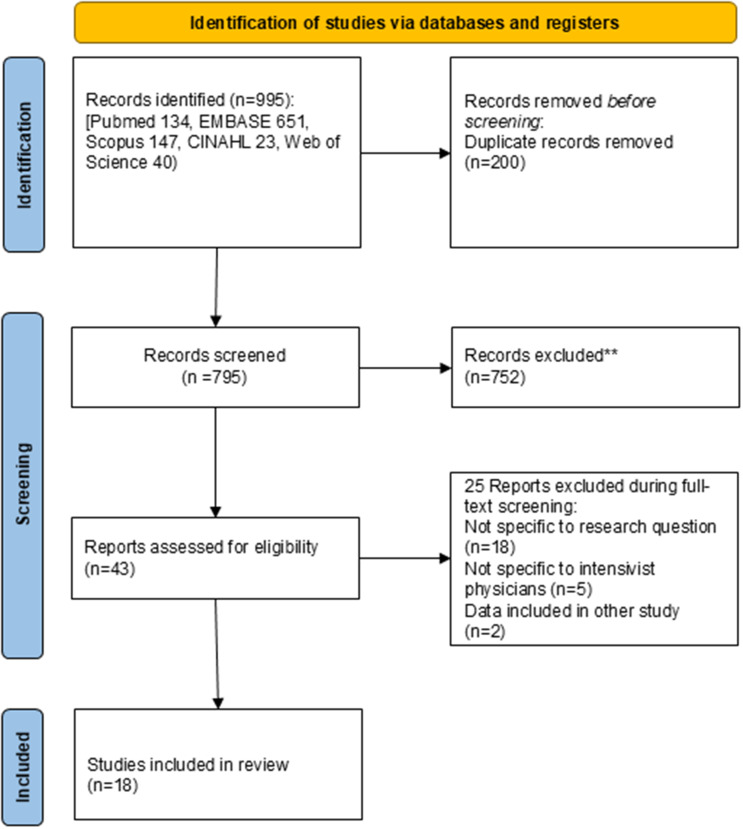
PRISMA 2020 flow diagram illustrating the study selection process for the scoping review.

The search retrieved 995 studies. After removing duplicates, 795 reports were screened of which title and abstract screening identified 43 studies for full-text review. After this step, a total of 18 studies were included in the review.^16–33^ The results of the study screening process are presented in the PRISMA diagram in Figure 1.

### Study characteristics

Most of the studies (55%, 10/18) were published in the last 5 years (2020–2024). The studies were mainly concentrated in and Europe (6) and North America (6), with the remaining distributed across Asia and South Pacific (3), Africa (1), and multicenter studies covering multiple continents (2).

Fourteen studies are quantitative, cross-sectional surveys. Four studies are qualitative and used semi-structured interviews. According to their objectives, studies were divided into those that address KAP related to antimicrobial prescribing behavior (13) and those that address perceptions of the impact of bacterial resistance (5).

In six studies, the evaluated population was mixed, with five including various medical specialties and one involving both intensive care physicians and nurses. In all of these, data from intensive care physicians and other categories are presented independently. The remaining 11 studies included only intensive care physicians as participants; three involved pediatric intensivists and one involved neonatologists (Table 1).

**Table 1.** Summary of characteristics of included studies in the scoping review of knowledge, attitudes, and practice

#	Year	First author	Country	Design	Study population	Sample/Response rate	Other professionals included
1	2001	Sintchenko, V.	Australia	Cross-sectional survey	Mixed	80/128 (62,5%)	Infectious disease physician
2	2009	Lepape, A.	European multicenter	Cross-sectional survey	Mixed	95/NA	NA
3	2010	Patel, SJ	USA	Cross-sectional survey	NICU	53/NA	NA
4	2015	Paño-Pardo, JR	Spain	Cross-sectional survey	Pediatric	114/206 (55,3%)	NA
5	2016	Steinberg, M.	Canada	Cross-sectional survey	Adult	185/634 (29%)	NA
6	2018	Gupte, V.	India	Cross-sectional survey	NR	539/539 (100%)	NA
7	2019	Evans, B	Canada	Cross-sectional survey	NR	15/22 (68,2%)	NA
8	2019	Rello, J.	Multicenter	Cross-sectional survey	Adult	63/NA	Infectious disease physician
9	2020	Noël, KC	Multicenter	Cross-sectional survey	Pediatric	482/1,251 (39%)	NA
10	2020	Rynkiewich, K	USA	Ethnographic observation	Adult	10/NA	NA
11	2020	Lepape, A.	European multicenter	Cross-sectional survey	Mixed	1,062/NA	NA
12	2021	Liu, J	China	Cross-sectional survey	Adult	3,687/3,789 (97,3%)	NA
13	2022	Fontela, P	Canada	Semi structured interview	Pediatric	21/NA	NA
14	2022	Shendy, EM	Egypt	Cross-sectional survey	Mixed	92/92 (100%)	NA
15	2022	Pandolfo, AM	England	Semi structured interview	Mixed	341/NA	NA
16	2023	Almeida-Costa	Portugal	Cross-sectional survey	Adult	33/40 (82%)	General surgery and internal medicine
17	2023	Rynkiewich, K	USA	Ethnographic observation	Adult	16/NA	NA
18	2024	Zainaghi et al	Italy	Cross-sectional survey	Mixed	29/21,3%	ICU nurses

The sample size ranged from 10 to 3,687 (median = 93), considering only the intensive care physicians in studies with mixed participants. Among the quantitative studies with a predefined sample size (*n* = 10), the average response rate was 65% (21.1–100%).

## Data collection instruments

The use of theoretical frameworks to build questionnaires and semi-structured interviews was described in only 3 studies,^24,31,32^ with the following theories applied: The COM-B system (Capability, Opportunity, Motivation—Behavior) (1),^24^ Necessity Concerns Framework (1),^31^ and Theory of Planned Behavior (1).^32^ For most studies, the composition of questionnaires was based on a literature review of similar previous studies and expert opinion. In five studies,^16,24,25,29,30^ the questionnaire went through a content and face validation process before being applied, and in two of these, the survey was adapted from another study, with validation occurring in the original study. In seven studies^16,24–27,30,32^ there was a pilot test, and one study^16^ employed a retest. Four studies^24–26,32^ were written in the local language and provided an English version of the questionnaire. The number of items per questionnaire ranged from 20 to 55, with five studies^16,18,21,25,26^ not reporting this information. In most cases, a commercial platform was used to host the questionnaire, with the link distributed via e-mail. In one study,^27^ conducted in 1999, the paper questionnaire was sent by mail (Table 2).

**Table 2.** Characteristics of measurement instruments

#	First author (Year)	Language of instrument	Theoretical framework	Development process	Content validity	Face validity	Pilot study	Retest	Number of related items	Assessed theme	Tool (Send by)
1	Sintchenko (2001)	English	NR	NR	NR	NR	Yes	NR	55	Attitudes regarding antibiotic use	Paper (Mail)
2	Lepape (2009)	NR	NR	NR	NR	NR	NR	NR	NR	Perception of antibiotic resistance and experience with infections by listed resistant bacteria	NR (Available in ESICM website)
3	Patel (2010)	English	NR	NR	NR	NR	NR	NR	43	Knowledge, attitude, and practices with regard to antimicrobial resistance and antimicrobial stewardship	NR
4	Paño-Pardo (2015)	Spanish	NR	Literature review of similar studies and expert opinion	NR	NR	Yes	NR	NR	Attitudes and perceptions regarding antimicrobial resistance and antimicrobial use	Navandú (e-mail)
5	Steinberg (2016)	English	NR	Focus group sessions and expert opinion	Yes	Yes	Yes	Yes	NR	Knowledge, attitude, and experiences on antimicrobial stewardship	SurveyMonkey (e-mail)
6	Gupte (2018)	English	NR	Focus group sessions and expert opinion	Yes	Yes	NR	NR	28	Perception and practice on antimicrobial resistance	NR
7	Evans (2019)	English	NR	Expert opinion	NR	NR	NR	NR	NR	Attitudes and perceptions on antimicrobial stewardship	SurveyMonkey (e-mail)
8	Rello (2019)	English	NR	Expert opinion	NR	NR	NR	NR	35	Knowledge, perceptions, and practices on antimicrobial resistance	SurveyMonkey (e-mail)
9	Noël (2020)	English, French, Portuguese, Japanese and Italian	NR	Literature review and specialist focus group sessions	Yes	Yes	Yes	NR	NR	Knowledge and practice in antibiotic prescribing	LimeSurvey (e-mail)
10	Lepape (2020)	English	NR	NR	NR	NR	NR	NR	20	Perception of antibiotic resistance and experience with infections by listed resistant bacteria	NR (Available in ESICM website)
11	Shendy (2022)	English	NR	Literature review	Yes*	Yes*	NR	NR	29	Attitudes and perceptions on antibiotic prescriptions and antibiotic resistance	NR (e-mail)
12	Almeida-Costa (2023)	Portuguese	Theory of Planned Behavior	Literature review	NR	NR	Yes	NR	50	Knowledge, attitude, and practice on antimicrobial prescription	Google Forms (sent to the head of the departments)
13	Zainaghi (2024)	Italian	COM-B	Adapted from a prior survey	Yes*	Yes*	Yes*	NR	26	Knowledge, attitude, and practice on antimicrobial resistance	Google Forms (sent to the head of the departments)

* Adapted from a previous study, validity tested in the original study.

In three of the four qualitative studies, the data collection method adopted was an ethnographic observation of participants within the ICU environment, followed by semi-structured interviews. The other study used focus groups and interviews based on clinical vignettes. The data collection period ranged from 2 to 24 months (Table 3).

**Table 3.** Characteristics of qualitative studies

#	First author (Year)	Data collection method	Data analysis method	Data collection period	Setting
1	Rynkiewich (2020)	Participants observation and semi-structured interview	Thematic coding	2 months	Surgical intensive care units in two different hospitals. In total, 4 SICU attending surgeons, 2 SICU attending anesthesiologists, one SICU attending pulmonologist, 2 surgery fellows, and 1 pulmonology fellow were observed,
2	Fontela (2022)	Participants observation and semi-structured interview	Grounded theory	24 months	Pediatric intensive care units in three different hospitals. The observed group include: PICU staff and fellows, junior and senior staff, male and female physicians
3	Pandolfo (2022)	Focus group and vignette-based interview (VBI)	Deductive approach	5 months	Pediatric and General intensive care units in four different hospitals. In total, 26 participants in focus groups, and 34 participants in VBI
4	Rynkiewich (2023)	Participants observation and semi-structured interview	Inductive approach	6 months	Medical intensive care units in two different hospitals. In total, 8 lead physicians, 8 physician fellows, 30 physician residents, and 2 specialty pharmacists were observed

## KAP toward antibiotic use and perception of the impact of resistance

### Knowledge

Knowledge was assessed differently across studies, through direct questions about resistance or scenarios indicating the use of antibiotics.^19,21–29,31,32^ In one study, it was assessed through clinical vignettes.^25^ Considering the set of included studies, few domains of antibiotic knowledge were assessed, with topics such as pharmacokinetics-pharmacodynamics, spectrum, indications, and resistance mechanisms not being addressed. However, this reflects the boundaries of the review scope, as studies evaluating highly specific or technical knowledge domains were not included.

Fontela *et al*. proposed a model of the physician’s decision-making process regarding antimicrobial prescribing, with the initial step relying on objective criteria, which largely depend on the knowledge acquired about antibiotic use.^19^

In five studies,^21–23,26,29^ the perception of prescribing physicians regarding the most concerning locally prevalent multidrug-resistant bacteria was assessed. In all of them, *Extended-Spectrum β-Lactamase*-producing *Enterobacterales* were identified as the most relevant. None of the studies compared the adequacy of physicians’ perceptions with local microbiological data. In the study by Zanaighi et al., 95.8% of professionals knew what antibiotic resistance was, and 100% of physicians demonstrated awareness that antibiotics are ineffective against viruses.^24^ Similarly, the study by Almeida-Costa et al showed that all physicians knew that inappropriate antibiotic use increases the incidence of AMR.^32^ The study by Liu et al specifically evaluated knowledge on prolonged infusion of antimicrobials and reported nearly 95% of intensivists were familiar with the concept of prolonged antibiotic infusion.^28^

However, knowledge gaps were also observed in other areas or instance, in the study by Sintchenko et al., nearly half of the physicians considered treating a patient with ventilator-associated pneumonia and a positive blood culture for coagulase-negative *Staphylococci* ^*27*^. Similarly, in the study by Zainaghi, almost 14% of physicians were unaware that healthy individuals can carry resistant bacteria.^24^

### Attitudes

The assessment of attitudes in the studies was generally done through questions that investigated beliefs and perceptions about antimicrobials and resistance.^16–20,24,26,27,30–33^ In the study by Fontela et al., the model of the therapeutic decision-making process shows a strong influence by attitude factors such as individual tolerance to uncertainty and the prescriber’s intuition, shaped by previous experiences.^19^ Pandolfo et al observed these factors, which include beliefs related to patient safety, the physician’s legal security, and the physician’s public image among peers.^31^

Another important attitude described in the included studies was that prescribing physicians tend to view the decision-making process for antimicrobial prescriptions as straightforward,^32^ feel confident in executing it,^24^ and believe that the loss of autonomy in prescribing can lead to frustration for intensivists when dependent on approval from another physician, as observed in the setting of an open surgical ICU.^17^ Conversely, specialists outside the ICU can also negatively influence excessive antimicrobial use.^19,31^

In two studies, more than fifty percent of the intensive care physicians perceived that the ASP positively changed their antimicrobial prescription.^16,18^ One study indicated that intensivists working in hospitals with an implemented ASP reported higher confidence in their antimicrobial prescriptions compared to those in institutions without a program.^30^

Although intensivists are aware of the locally prevalent resistance profiles in their setting, as mentioned above, they viewed the development and spread of resistance as more distant from their own prescribing practices. In the study by Almeida-Costa et al., 45% of intensivists did not perceive their prescriptions as contributing to AMR.^32^ The study of Pandolfo et al demonstrated that physicians saw antibiotics as preventing consequences for which they are immediately responsible. In this context, concerns regarding the negative effects of antimicrobial use at the individual or collective level were rarely prioritized.^31^ Similarly, intensivists considered that the local resistance pattern was relevant information, yet the argument of side effects and resistance development did not influence their decision-making.^19,27^ On the other hand, prescription decisions were influenced by internal factors as physicians’ concerns about the consequences of avoiding use of antibiotics, past experience, and clinician capability, and environmental context, such as pressure from consultants^31^ and hierarchical relationships.^16^

### Practice

The practice was assessed in eight studies.^24–27,29,30,32,33^ The use of guidelines as a reference by prescribing physicians was assessed in various ways across the studies included in this review. In the study by Zainaghi et al., 96% of physicians reported trusting antimicrobial therapy guidelines.^24^ The study by Sintchenko et al highlighted that local guidelines are considered an important tool by physicians,^27^ while the study by Shendy et al found that intensivists consider them more useful than international guidelines.^30^ Three studies evaluated whether prescribers know where to find institutional protocols, with results ranging from 69% to 86%.^24,30,32^ Adherence to protocols was assessed in one study through self-evaluation, in which 87% of intensivists reported regularly following local guidelines.^26^

Obtaining microbiological samples before initiating antimicrobials is a common practice. However, practices not supported by evidence were reported in some studies. In the study by Gupte et al., up to 41% of intensivists preferred continuing the initial carbapenem treatment, regardless of the susceptibility report, in patients showing clinical improvement.^29^ In another study, using vignettes, 16%–68% of pediatric intensivists, across different countries, did not discontinue antibiotics even after a diagnosis of a viral illness was established.^25^

## Discussion

This scoping review synthesizes the main findings on the KAP of intensive care physicians regarding antimicrobial use and AMR. Our study found that knowledge remains relatively unexplored, with most assessments focusing primarily on understanding the relationship between antibiotic use and resistance. However, we only included studies that evaluated antimicrobial use KAP in a general sense, not those addressing specific subtopics. As such, more in-depth concepts essential for the appropriate use of antimicrobials are rarely assessed or not evaluated at all, leaving a gap in mapping that could inform future interventions. Attitudes play a crucial role in the decision-making process for antimicrobial prescribing and in shaping the perception of resistance. Meanwhile, prescribing practices, influenced by both factors, demonstrated strong physician acceptance of guidelines, though without clear evidence of adherence. Additionally, few studies used theories and frameworks, which hinders the reproducibility and comparability of results.

The decision-making process for prescribing antimicrobials is complex, involving knowledge and, as proposed by Warreman et al., influenced by both internal and external factors affecting the prescribing physician.^10^ These include sociodemographic elements, previous experiences, tolerance to uncertainty, emotions, and interactions with others, such as the patient, the pharmaceutical industry, and medical peers.^10,34^ For this reason, studies evaluating the determinants of antimicrobial prescribing should incorporate aspects of social sciences and psychology to better understand prescribing behavior.^35–37^

In the context of attitudes that influence the prescription of antimicrobials, factors such as concern about the risk of patient deterioration stand out, representing both a clinical risk and a protective factor for the physician in the legal sphere. These elements are linked to tolerance to uncertainty. A clear diagnosis is an objective tool for guiding rational antimicrobial use, but it cannot always be established. Diagnostic uncertainty may contribute to increased prescription of antimicrobials in various clinical scenarios.^38,39^ Uncertainty is part of the human condition and is present in numerous healthcare settings, arising from various unknowns, such as patient progression, response to antimicrobials, and the causative agent of infection, among others. Uncertainty can evoke fear, anxiety, and hesitation, impacting therapeutic decision-making. The response to uncertainty is variable and may affect the quality of care provided. Managing uncertainty is complex. Understanding these responses and how physicians tolerate uncertainty can help guide strategies to mitigate it, such as advancements in rapid diagnostics and handshake stewardship.^18,40,41^

Prescribing practices result from intrinsic factors such as knowledge and attitudes, combined with extrinsic factors like the healthcare system, relationships with patients, and interactions with medical peers.^34^ In survey studies, information about practices may carry the bias of being reported based on how physicians perceive their prescribing patterns rather than through direct observation by the researcher. Nevertheless, some discrepancies can still be observed, such as the failure to de-escalate antibiotics despite positive culture results and the continued use of antibiotics in cases of diagnosis of viral etiology.^25,29^ Another important aspect is that intensivists prefer local guidelines over national or international ones for antimicrobial prescribing. This same trend is noted in other systematic reviews.^42,43^ However, although intensivists express greater benefit from local protocols in their practice, studies evaluating adherence to institutional recommendations demonstrate low compliance,^44,45^ which may result from influencing factors such as perceived restriction of autonomy, disagreement with recommendations, and lack of motivation.^46^

In addition to guidelines and the need for adherence, the prescribing physician needs to be aware of local resistance patterns to the appropriate selection of empirical antibiotics. In a systematic review, an average of 39.6% of respondents were aware of local resistance patterns, and physicians frequently perceived resistance as a distant problem from their reality.^42^ On the other hand, in the studies from our review that evaluated this aspect, intensivists reported the presence of MDR bacteria in their setting; however, they have a limited understanding of the relationship between resistance incidence and their prescribing practices. Furthermore, intensivists consider the strategy of correlating individual-level prescribing with the development of resistance to be of limited effectiveness as an ASP intervention, as this is rarely prioritized over the risk of poor clinical outcomes for the patient.

The ICU is commonly prioritized in AMS initiatives. For this reason, understanding intensivists’ KAP regarding antimicrobial prescribing is essential to guide interventions. However, a systematic review of 19 studies^43^ conducted in different countries with physicians from various specialties reported findings like those in our study, suggesting that there may not be a significant difference between intensivists and non-intensivists.

Survey studies are an important research method for generating questions that will be explored in other types of studies. Therefore, they require rigor in their design and analysis to ensure the reliability of the generated data, reproducible, and unbiased. As a strategy for collecting information about practices, attitudes, and beliefs, questionnaires may have limitations compared to interviews and focus groups, which yield more detailed information. However, they offer advantages in their simpler application, particularly with the online availability of questionnaires,^47^ making them an increasingly used mode of research.

The elaboration of questionnaires and interviews in these studies was usually based on literature reviews and expert opinion, except for three studies that used a theory or theoretical framework. A theory is defined as the relationship between concepts that help understand a broader picture, while a theoretical framework is developed from one or more theories and connects a set of concepts and premises to support a study.^48^ Theories and frameworks provide a structure that facilitates the understanding and generalization of results. Moreover, questions developed from psychological theories allow for a depth of inquiry that questionnaires based solely on literature reviews cannot achieve. Thus, future research should address the underutilization of theories and frameworks by incorporating them into questionnaire development, thereby enhancing the quality of survey studies and advancing implementation science.^35^

In addition to using theories and frameworks for developing constructs, the questionnaire development and validation process should follow a rigorous and systematic flow.^36,37^ However, few studies described the implementation of a questionnaire validation process, resulting in a certain degree of fragility regarding the quality and reproducibility of the questionnaires.

Based on the gaps identified in this scoping review, several avenues for future work emerge. First, there is a need for studies employing standardized and validated KAP instruments to allow meaningful comparisons across settings and regions. Second, future research should explore how specific behavioral determinants influence prescribing decisions in the ICU using established implementation science frameworks. Finally, future studies should ensure broader geographic representativeness, as current evidence is concentrated in a limited number of countries and regions, limiting the generalizability of findings.

To the best of our knowledge, this is the first review focused on mapping KAP among intensive care physicians. This is significant because the ICU represents the setting with the highest density of antimicrobial use in hospitals, and the determinants of antimicrobial prescribing behavior among intensivists tend to differ from those of general physicians. This study also evaluated the construction of the questionnaires used in the primary studies, allowing for inferences regarding the quality and generalizability of the information obtained.

This study has some limitations. The number of studies that met the inclusion criteria was small and lacked representation from all regions of the world, which limits the generalizability of the conclusions. There was heterogeneity in the methods and assessment approaches, as well as in the psychometric properties of the questionnaires. In addition, our decision to include only studies that broadly assessed antimicrobial prescribing KAP, while excluding those focused on specific topics, may have limited the scope of the findings. Finally, because we did not apply any time restriction during the literature search, some included studies reflect older resistance patterns and prescribing practices, which may differ from current clinical realities.

In conclusion, the decision-making process for antimicrobial prescribing is complex and should always take behavioral factors into account for more effective interventions. Therefore, the assessment of KAP serves as a strategic tool. The evaluation of intensivists’ KAP regarding antimicrobial prescribing and their perception of bacterial resistance reveals significant opportunities for intervention in the components of attitudes and practices, as well as a better understanding of the impact of resistance at the local and individual levels, with the ASP being a potential facilitator in this process.

## Supporting information

10.1017/ash.2025.10249.sm001 Piastrelli et al. supplementary material Piastrelli et al. supplementary material

## Supplementary material

The supplementary material for this article can be found at https://doi.org/10.1017/ash.2025.10249.
