# Supplementary material for: Knowledge, attitudes, and practices of ICU physicians on antimicrobial use and resistance: a scoping review
Source: Antimicrob Steward Healthc Epidemiol. 2025 Dec 18;6(1):e1. doi: 10.1017/ash.2025.10249 (PMC12766526; doi:10.1017/ash.2025.10249)
Supplement: Piastrelli et al. supplementary material [file S2732494X25102490sup001.docx]

# Supplementary File. Complete Search Strategies

## 1. PubMed (20 Nov 2025)

(Physicians[MeSH] OR physician*[tiab] OR doctor*[tiab] OR intensivist*[tiab] OR neonatologist*[tiab])
AND
("health knowledge, attitudes, practice"[MeSH] OR "practice patterns, physicians"[MeSH] OR "attitude of health personnel"[MeSH] OR "knowledge attitude practice"[tiab] OR "knowledge and attitude"[tiab] OR perception*[tiab] OR belief*[tiab] OR behavio*[tiab] OR determinant*[tiab] OR "facilitator* and barrier*"[tiab])
AND
("Anti-Bacterial Agents"[MeSH] OR "drug resistance, bacterial"[MeSH:noexp] OR "antibiotic prescription"[tiab] OR "antibiotic us*"[tiab] OR "antimicrobial prescription"[tiab] OR "antimicrobial us*"[tiab] OR "antibiotic utilization"[tiab] OR "antimicrobial utilization"[tiab] OR "antibiotic resistance"[tiab] OR "antimicrobial resistance"[tiab])
AND
("Intensive Care Units"[MeSH] OR "intensive care units, neonatal"[MeSH] OR "intensive care units, pediatric"[MeSH] OR "intensive care unit*"[tiab] OR "intensive care"[tiab] OR "critical care"[tiab] OR "intensive care medicine"[tiab] OR "critical care medicine"[tiab] OR "critical care unit*"[tiab])

## 2. Embase (20 Nov 2025)

('physician'/exp OR physician:ti,ab OR doctor:ti,ab OR intensivist:ti,ab OR neonatologist:ti,ab)
AND
(attitude/exp OR 'health personnel attitude'/exp OR 'knowledge attitude practice':ti,ab OR 'knowledge and attitude':ti,ab OR perception*:ti,ab OR belief*:ti,ab OR behavio*:ti,ab OR determinant*:ti,ab OR 'facilitator* and barrier*':ti,ab)
AND
('antiinfective agent'/exp OR 'antibiotic resistance'/exp OR 'antibiotic prescription':ti,ab OR 'antibiotic us*':ti,ab OR 'antimicrobial prescription':ti,ab OR 'antimicrobial us*':ti,ab OR 'antibiotic utilization':ti,ab OR 'antimicrobial utilization':ti,ab OR 'antibiotic resistance':ti,ab OR 'antimicrobial resistance':ti,ab)
AND
('intensive care unit'/exp OR 'neonatal intensive care unit'/exp OR 'pediatric intensive care unit'/exp OR 'intensive care unit*':ti,ab OR 'intensive care':ti,ab OR 'critical care':ti,ab OR 'intensive care medicine':ti,ab OR 'critical care medicine':ti,ab OR 'critical care unit*':ti,ab)

## 3. Scopus (20 Nov 2025)

(TITLE-ABS-KEY(physician OR doctor OR intensivist OR neonatologist))
AND
(TITLE-ABS-KEY("health personnel attitude" OR "knowledge attitude practice" OR "knowledge and attitude" OR perception* OR belief* OR behavior* OR determinant* OR "facilitators and barriers"))
AND
(TITLE-ABS-KEY("antiinfective agent" OR "antibiotic resistance" OR "antibiotic prescription" OR "antibiotic use" OR "antimicrobial prescription" OR "antimicrobial use" OR "antibiotic utilization" OR "antimicrobial utilization" OR "antimicrobial resistance"))
AND
(TITLE-ABS-KEY("intensive care unit" OR "neonatal intensive care unit" OR "pediatric intensive care unit" OR "intensive care" OR "critical care" OR "intensive care medicine" OR "critical care medicine" OR "critical care unit"))

## 4. CINAHL (20 Nov 2025)

((TI(physician* OR doctor* OR intensivist* OR neonatologist) OR AB(physician* OR doctor* OR intensivist* OR neonatologist)))
AND
((TI("Health Knowledge, Attitudes, Practice" OR "Practice Patterns, Physicians" OR "Attitude of Health Personnel" OR "knowledge attitude practice" OR "knowledge and attitude" OR perception* OR belief* OR behavio* OR determinant* OR "facilitator* and barrier*")
OR
AB("Health Knowledge, Attitudes, Practice" OR "Practice Patterns, Physicians" OR "Attitude of Health Personnel" OR "knowledge attitude practice" OR "knowledge and attitude" OR perception* OR belief* OR behavio* OR determinant* OR "facilitator* and barrier*")))
AND
((TI("Anti-Bacterial Agents" OR "Drug Resistance, Bacterial" OR "antibiotic prescription" OR "antibiotic us*" OR "antimicrobial prescription" OR "antimicrobial us*" OR "antibiotic utilization" OR "antimicrobial utilization" OR "antibiotic resistance" OR "antimicrobial resistance")
OR
AB("Anti-Bacterial Agents" OR "Drug Resistance, Bacterial" OR "antibiotic prescription" OR "antibiotic us*" OR "antimicrobial prescription" OR "antimicrobial us*" OR "antibiotic utilization" OR "antimicrobial utilization" OR "antibiotic resistance" OR "antimicrobial resistance")))
AND
((TI("Intensive Care Unit*" OR "Neonatal Intensive Care Unit*" OR "Pediatric Intensive Care Unit*" OR "intensive care" OR "critical care" OR "intensive care medicine" OR "critical care medicine" OR "critical care unit*")
OR
AB("Intensive Care Unit*" OR "Neonatal Intensive Care Unit*" OR "Pediatric Intensive Care Unit*" OR "intensive care" OR "critical care" OR "intensive care medicine" OR "critical care medicine" OR "critical care unit*")))

## 5. Web of Science (20 Nov 2025)

TS = (physician* OR doctor* OR intensivist* OR neonatologist*)
AND
TS = ("Health Knowledge, Attitudes, Practice" OR "Practice Patterns, Physicians" OR "Attitude of Health Personnel" OR "knowledge attitude practice" OR "knowledge and attitude" OR perception* OR belief* OR behavio* OR determinant* OR "facilitator* and barrier*")
AND
TS = ("Anti-Bacterial Agents" OR "Drug Resistance, Bacterial" OR "antibiotic prescription" OR "antibiotic us*" OR "antimicrobial prescription" OR "antimicrobial us*" OR "antibiotic utilization" OR "antimicrobial utilization" OR "antibiotic resistance" OR "antimicrobial resistance")
AND
TS = ("Intensive Care Unit*" OR "Neonatal Intensive Care Unit*" OR "Pediatric Intensive Care Unit*" OR "intensive care" OR "critical care" OR "intensive care medicine" OR "critical care medicine" OR "critical care unit*")
